# Large-scale data from wearables reveal regional disparities in sleep patterns that persist across age and sex

**DOI:** 10.1038/s41598-019-40156-x

**Published:** 2019-03-04

**Authors:** Ju Lynn Ong, Jesisca Tandi, Amiya Patanaik, June C. Lo, Michael W. L. Chee

**Affiliations:** Centre for Cognitive Neuroscience, Duke-NUS Medical School, Singapore, 169857 Singapore

## Abstract

Prior reports on geographical differences in sleep duration have relied on samples collected at different time points with a variety of subjective instruments. Using sleep data from a total of 553,559 nights from 23,680 Fitbit users (aged 15–80y), we found objective evidence for regional disparities in sleep duration of 32–43 min between Oceanian and East Asian users on weekdays. This was primarily driven by later bedtimes in East Asians. Although users in all countries extended sleep on weekends, East Asians continued to sleep less than their Oceanian counterparts. Women generally slept more than men, and older users slept less than younger users. Reasons for shorter sleep duration in East Asians on both weekdays and weekends, across the lifespan and in both sexes remain to be investigated.

## Introduction

Voluntary sleep restriction is a global epidemic, with widespread consequences on health, safety and productivity. Short sleepers (<7 h/night) present a 12% greater risk of all-cause mortality compared to those sleeping 7 to 8 h per night^1^. Furthermore, econometric modeling has showed that for five countries in the Organisation for Economic Co-operation and Development (OECD) losses of up to $680 billion a year can be attributed to insufficient sleep^2^.

Striking geographical differences in the extent of sleep loss have been documented in questionnaire-based studies^3–5^. East Asians sleep less and go to bed later than their Western counterparts^3–6^, likely due to longer working hours, intense pressure to excel, and an “always-connected” culture prevalent in Asia. In addition, these disparities in sleep duration appear to differ by age and sex. South Korean 12^th^-graders have been reported to average just 4.9 h of sleep a night^7^, compared to their peers in Australia who sleep 8.5 h (males) and 9.1 h (females)^8^. This discrepancy, while still present, was almost halved in older adults >65y, with studies reporting a mean of 6.5 h in South Korea^9^ and 8.3 h in Australia^10^.

Prior reports comparing countries are predominantly based on comparisons of single country studies that were collected using a variety of subjective instruments and time windows instead of head-to-head comparisons using objective instruments. As such there is merit in establishing the implied geographical differences in sleep duration using large scale actigraphy. Self-report measures, while easily scalable, can be unreliable^11^, being subject to rounding errors and recall bias, as well as instrument-related differences. For example, data collected from single-question surveys demonstrate poor agreement (within ± 2.5 h) with data collected using 24 h time-use surveys^12^.

The influx of inexpensive, consumer-based activity trackers in this era of big data present an unprecedented opportunity to objectively characterize sleep habits on a global level. In 2017 alone, worldwide wearable device shipments reached a 115 million units^2^. These trackers would also allow for the standardized collection of data from populations with limited reporting ability, for example in young children, older adults, or clinical populations, and across different language groups.

In the present study, we analyzed large-scale sleep data from Fitbit users in five countries located within two geographical regions: Oceania (Australia and New Zealand) and East Asia (Singapore, Hong Kong and South Korea), in order to quantify cross-country differences in objective sleep patterns. These regions include countries typically at either end of the sleep duration spectrum^4^. We also investigated whether these disparities in sleep patterns differed by age group and sex. In addition, as weekday and weekend sleep patterns typically differ^5,6,13^, we conducted separate analyses for both day types, as well as for weekday-weekend sleep extension.

## Results

Anonymized sleep data from 23,680 Fitbit users aged 15–80 over the period 1^st^ March 2017 to 1^st^ April 2017 contributed to this report. Users were randomly selected from the Fitbit user-base in Australia, New Zealand, Singapore, Hong Kong and South Korea. The sample consisted of more female users in all countries (52–75%; Fig. S1) except in South Korea (40% female). In the East Asian samples, there were also fewer 15–20y, 66–70y and 71–80y users (<5%) relative to the other age groups (5–13%), compared to the Oceanian samples, where the distribution was roughly equal across all age groups (8–11%).

### Sleep duration differed by country on both weekdays and weekends

Country, age group and sex significantly influenced both weekday and weekend sleep duration (Ps < 0.001, Table S1). Of these, country was the largest determinant of sleep duration (η_p_^2^ = 0.04–0.07; Table S1), with effect sizes outweighing those of age group and sex (η_p_^2^ = 0.01; Table S1).

On weekdays, there were striking differences in sleep duration between Oceanian and East Asian countries (32–43 min). More than half of the population in Oceania (Australia: 61%, New Zealand: 66%) received at least 7 h of sleep, compared to a less than a third in East Asia (Singapore: 27%, Hong Kong: 34%, South Korea: 29%). Differences in bedtimes were the main driver of differences in sleep duration. Users in East Asian countries slept on average, 59–84 min later (Ps < 0.001; Table S2) than users in Oceanian countries. Despite their later bedtimes East Asian users woke up only slightly later, on average 18–50 min (Ps < 0.001; Table S2).

Regional differences in sleep duration persisted into the weekend. Between-region differences were 22–42 min on average (Ps < 0.001; Table S2). Although the proportion of users obtaining at least 7 h of sleep increased on weekends, barely half of East Asians obtained 7 h of sleep or more (Singapore: 51%, Hong Kong: 58%, South Korea: 52%) compared to three-quarters of persons in Oceania (Australia: 74%, New Zealand: 79%). Similar to weekday sleep, this was due to later bed times (54–79 min; Ps < 0.001; Table S2) paired with relatively shorter delays in wake times (12–59 min; Ps < 0.001; Table S2) in the East Asian countries.

Overall, although there was a main effect of country on weekday-weekend sleep extension (P < 0.001; Table S1), numerical differences both within- and between-regions were relatively small (within-region: 3–9 min, between-region: 0–10 min; Table S2).

### Regional differences in sleep duration were observed in all age groups

**Figure 1:**
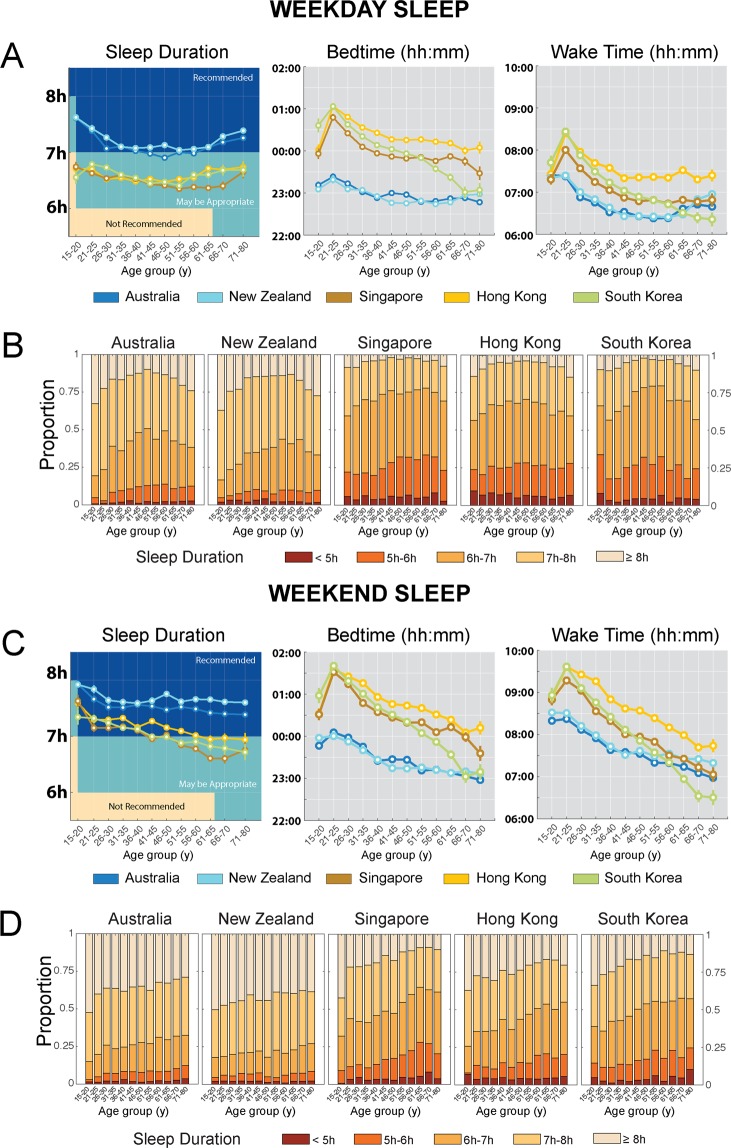
Regional differences in sleep patterns by age group. (**A**) Estimated marginal means and standard errors of sleep duration, bedtimes and wake times by country and age group for weekdays. Sleep duration recommendations from the National Sleep Foundation (14) are overlaid on sleep duration plots for comparison. (**B**) Proportion of users sleeping <5 h, 5–6 h, 6–7 h, 7–8 h and ≥8 h a night by country and age group on weekdays. (**C**) Estimated marginal means and standard errors of sleep duration, bedtimes and wake times by country and age group for weekends. Sleep duration recommendations from the National Sleep Foundation (14) are overlaid on sleep duration plots for comparison. (**D**) Proportion of users sleeping <5 h, 5–6 h, 6–7 h, 7–8 h and ≥8 h a night by country and age group on weekends. Large differences between Oceanian and East Asian users were consistently observed across all age groups on both weekdays and weekends.

On weekdays, a significant quadratic trend in sleep duration with age was found in all countries (Ps ≤ 0.007; Fig. 1A). Sleep duration reached a minimum between 46–55y of age. On weekends, age trends were more linear (Ps < 0.001; Fig. 1C), with sleep duration generally decreasing with increasing age.

Country differences in sleep duration were observed in all age groups. However, their magnitude differed by age group, as evidenced by significant country x age group interactions on both weekdays (F = 2.42, P = 0.001; Table S1) and weekends (F = 2.69, P = 0.001; Table S1).

On weekdays, between-region differences in sleep duration were in the order of 20–64 min (Ps < 0.001; Fig. 1A) with the largest difference occurring between New Zealand and South Korea in the 15–20y age group. On average, East Asian users in all age groups were obtaining less than the amount of sleep duration recommended by the National Sleep Foundation^14^ (Fig. 1A). These regional disparities were further reflected in the distribution of weekday sleep duration by country and age group in Fig. 1B. While 15–20 year-old users appear to be obtaining the most sleep on average, the recommended sleep duration is also the highest. Adjusting for a minimum sleep duration of 8 h in this age group revealed that a majority of users across all countries (Oceanian: 63–67%, East Asian: 86–92%) obtained insufficient sleep on weekdays. The proportion of short sleepers (<8 h in 15–20 year-olds vs. <7 h in the other age groups) was also greatest in this segment than in any other age group (Oceanian: 23–51%, East Asian: 57–79%).

East Asian users generally slept later on weekdays (30–104 min, Ps ≤ 0.004; Fig. 1A) compared to Oceanians of the same age. Although users in East Asian countries across most age groups surveyed also woke up later (13–65 min, Ps < 0.05; Fig. 1A) than similarly aged Oceanians, this difference was numerically smaller compared to the magnitude of delayed bedtimes.

Regional differences also persisted on the weekends. Between-region differences were 11–63 min (Ps < 0.05; Fig. 1C) with the largest differences occurring between New Zealand and Singapore in the 61–65y age group. Although both regions obtained more sleep on weekends, East Asian participants were either only barely obtaining or still not achieving the recommended amount of sleep (Fig. 1C). 57–66% of East Asian adolescent samples obtained <8 h of sleep on weekends, compared with 48–49% in the Oceanian samples (Fig. 1D). In the other age groups, 35–64% of the East Asian users obtained <7 h of sleep compared with only 18–32% of the Oceanian users (Fig. 1D).

On weekends, users in East Asian countries slept later than users in Oceanian countries in all age groups <66y (26–100 min, Ps < 0.001; Fig. 1C). Users in East Asian countries across age groups <51y also woke up later (14–81 min; Ps < 0.05; Fig. 1C) than their Oceanian counterparts; however, this difference was again numerically smaller compared to the magnitude of delayed bedtimes.

### Regional differences in sleep duration were found in both males and females

**Figure 2:**
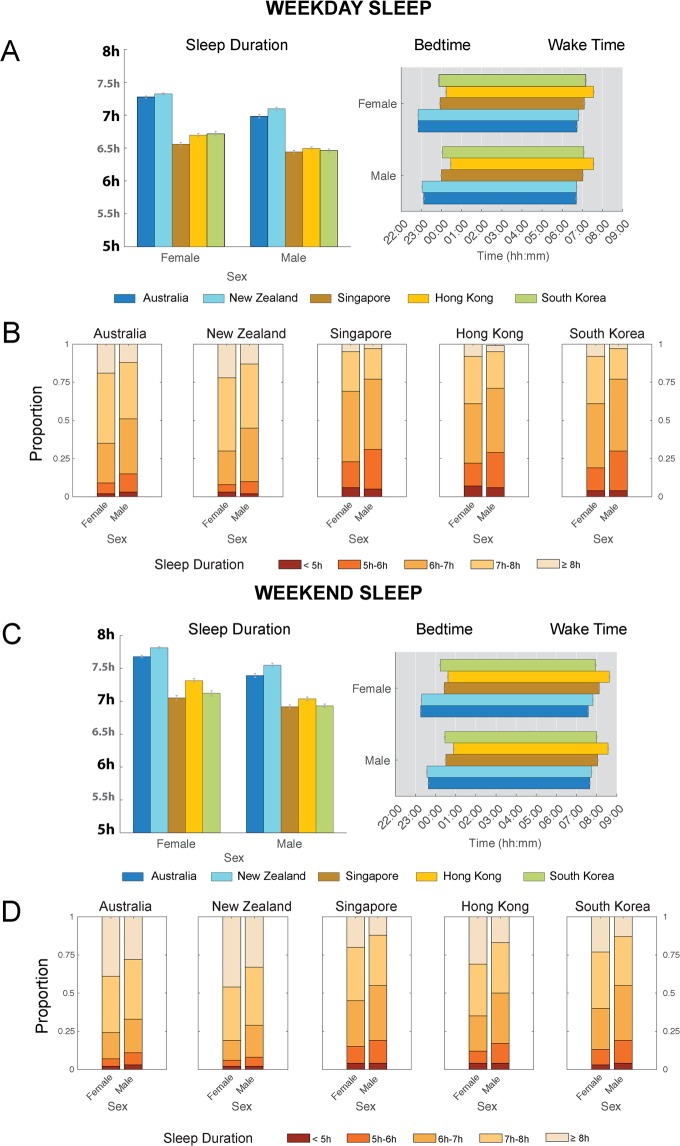
Regional differences in sleep patterns by sex. (**A**) Estimated marginal means and standard errors of sleep duration, bedtimes and wake times by country and sex for weekdays. (**B**) Proportion of users sleeping <5 h, 5–6 h, 6–7 h, 7–8 h and ≥8 h a night by country and sex on weekdays. (**C**) Estimated marginal means and standard errors of sleep duration, bedtimes and wake times by country and sex for weekends. (**D**) Proportion of users sleeping <5 h, 5–6 h, 6–7 h, 7–8 h and ≥8 h a night by country and sex on weekends. Large differences between Oceanian and East Asian users were consistently observed across both sexes on both weekdays and weekends.

In general, females slept more than males in all countries surveyed (Ps ≤ 0.001; Fig. 2). However, there was a significant country x sex interaction effect on both weekdays (F = 4.10, P = 0.003; Table S1) and weekends (F = 2.63, P = 0.03; Table S1). Within each sex, large between-region differences in weekday sleep duration were again observed, but more prominently in females (Males: 29–40 min; Females: 33–46 min; Ps < 0.001; Fig. 2A). For females, 61–69% East Asian users were obtaining <7 h of sleep, compared with 30–34% Oceanian users (Fig. 2B). For males, this number was increased, with 72–77% East Asian users obtaining <7 h of sleep compared with 45–77% Oceanian users (Fig. 2B). Large between-region differences in both sexes were also observed for bed times (Males: 53–85 min; Females: 62–84 min; Ps ≤ 0.001; Fig. 2A) and wake times (Males: 19–51 min; Females: 17–50 min; Ps ≤ 0.001; Fig. 2A).

On weekends, between-region differences in sleep duration persisted (Males: 21–38 min; Females: 22–46 min; Ps ≤ 0.001; Fig. 2C). Although the proportion of users obtaining <7 h of sleep decreased on weekends, there were still 35–45% East Asian females were obtaining <7 h of sleep, compared with 19–23% Oceanian females (Fig. 2D). This proportion was higher in males, where 50–55% East Asian males obtained <7 h of sleep compared with 29–33% Oceanian males (Fig. 2D). Between-region differences were also pronounced for both bed times (Males: 49–79 min; Females: 56–82 min; Ps < 0.001; Fig. 2C) and wake times (Males: 15–54 min; Females: 9–63 min; Ps ≤ 0.005; Fig. 2C).

### Small regional differences in weekday-weekend sleep extension were observed in users <41y

**Figure 3:**
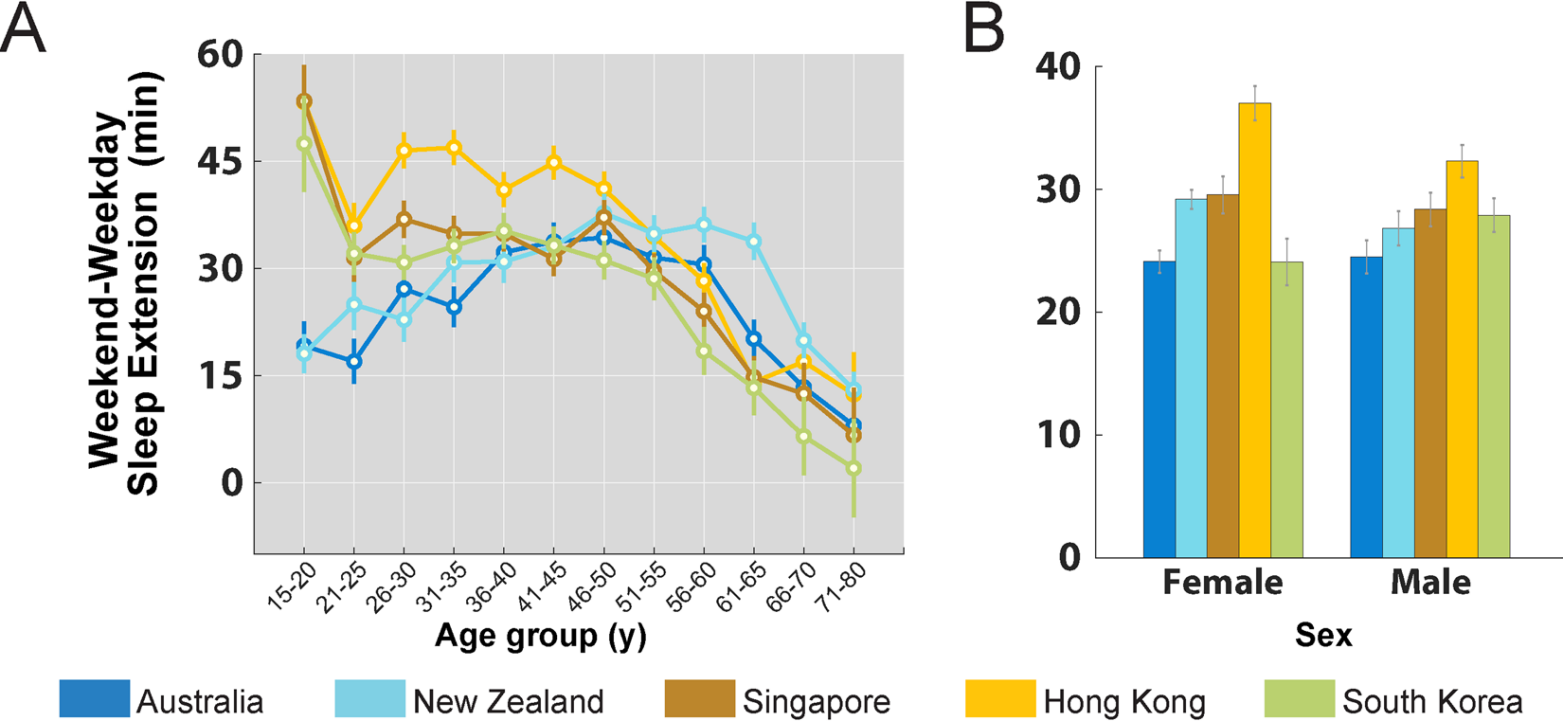
Weekday-weekend sleep extension. Estimated marginal means and standard errors of sleep extension (weekend – weekday sleep duration) by country and (**A**) age group, and (**B**) sex.

With the exception of South Korean users aged 66–80y, users in all age groups and countries extended sleep on the weekends by 8–54 min (one-sample t-tests against 0; Ps < 0.05; Fig. 3A). Although East Asian users <41y extended sleep more than their similarly aged Oceanians, between-region differences only ranged between 2–35 min, and was insufficient to allow East Asians to catch up with their Oceanian counterparts.

Sex differences in sleep extension however, did not differ by country (country x sex interaction P = 0.054; Fig. 3B).

## Discussion

Analysis of over 550,000 nights of data from East Asian and Oceanian (largely Westerners) users of a consumer sleep/activity tracker found significant country/region level differences in sleep duration, bedtime and wake times across the entire lifespan, considerably expanding on findings obtained from questionnaire data in narrower age bands^3,4^. Objectively measured sleep duration declined across the lifespan from adolescence to old age. Weekday sleep duration showed a quadratic trend across life and was overall shorter than weekend sleep which showed a linear downtrend. Women generally slept longer than their male counterparts. Sleep duration values were smaller in our study than those reported in prior work^3,4^, likely because subjective sleep measurements typically reflect time spent in bed, rather than time actually spent asleep. We elaborate on each of these in turn.

These differences observed are likely perpetuated by cultural differences in the two regions. The persistence of shorter sleep in East Asians throughout the lifespan, including the retirement years and in both sexes, speaks to the pervasive influence of culture on sleep habits. Perceptions of work-life balance vastly differ between eastern and western countries. Long work hours are often a yardstick of job commitment in Asia^15^. Although workers in Singapore had lower contracted hours than their Australian counterparts, they worked on average 12 hours/week over those hours^16^. In addition, owing to a strong emphasis on academic excellence, adolescents in Asia typically spend more time on schoolwork and private tuition as compared to their Western counterparts^7^. Both these work and educational demands reduce the amount of sleep one is able to obtain on weekdays. In addition, as short sleep duration appeared to be more strongly related to later bed times than wake times in the East Asian countries, policy makers^17^ should investigate reasons underlying later bed times and consider intervention strategies to promote going to bed earlier in order to increase time available for sleep.

The regional gap in sleep duration persisted on weekends, during which there is relative release from occupational/academic demands. This occurred even amongst younger East Asian users who presumably have the capacity to sleep longer. Either East Asians simply need less sleep, adapt to less sleep over time, or there exists a powerful cultural undervaluation of sleep^3^. The possibility that East Asians possess genetic variants that confer resilience to sleep loss has been suggested based on a study reporting similar levels of daytime sleepiness between Chinese and American schoolchildren despite less sleep in the former^18^. Even amongst Asians living in America, Asians were still more likely to report short sleep duration than Whites^19^. These studies should spur further evaluation of why Asians sleep less. While an alternative explanation is an adaptation to sleep loss over time, a recent laboratory-based study demonstrated that at least over the course of 1-week of chronic sleep restriction, homeostatic responses were preserved^20^. Whether adaptation occurs to longer periods of restriction is still undetermined.

While older adults tended to sleep less compared to young adults^21^, the magnitude of this difference differed on weekdays and weekends. On weekdays, there was a clear quadratic trend in sleep duration, with a trough occurring between the ages of 46–55. This ‘U-shaped’ pattern of sleep duration with age was more clearly expressed in the Westerner dominated Australia/New Zealand sample and is congruent with a prior U.S. based report that found an increase in sleep duration 15–22 min post-retirement^22^. Obligations to take care of grandchildren’s enrichment and tuition classes are common in extended family structures in East Asia and could contribute to the erosion of increased post-retirement sleep opportunity. On weekends, sleep duration decreased more linearly with age, likely reflecting a biological decline in sleep need/sleep capacity commonly described in studies of sleep and aging^23^.

A steep delay in bed/wake times on both weekdays and weekends was also observed in adolescents (15–20y) compared to young adults (21–25y). This pattern of sleep phase delay peaking around the early-mid 20 s has been demonstrated in prior work by Roenneberg and colleagues^17^. Interestingly, in the present study, the phase delay appears to be more prominent in the East Asian samples.

Sex differences in sleep duration have been mixed in the literature. Studies comparing samples averaged across regions or age groups generally find that females tended to report more sleep than males^24–26^. This observation was true even when considering objective polysomnographic measures^21^. In contrast, sex differences were non-significant in some countries^24^, when examining young or older single, childless respondents^25^, or when models were adjusted for socio-demographic and health variables^26^. In our sample, we found that averaged across all countries, sex effects on sleep duration were only significant in the 21–65 age range. This could indicate sex differences that only become distinct after graduation, and diminish after retirement.

A few limitations exist in this study. Motion-based trackers based on accelerometry are typically calibrated for use in healthy populations. Recent validation studies in adolescents^27^ (12–21y) and healthy adults^28^ (19–61y) found that the Fitbit Charge HR^TM^/Fitbit Charge 2^TM^ showed high sensitivity for sleep detection (96–97%) and only slightly overestimated TST by 8–9 min compared to gold-standard polysomnography measures. However, accuracies are likely to be lower for individuals with sleep disorders and those with a higher proportion of wake after sleep onset due to poorer specificity for wake detection in these devices^29^. A second limitation concerns representativeness of the sample. Users who purchase wearables typically fall in the medium to high socio-economic (SES) range, and are generally concerned about their health. SES has been shown to significantly impact sleep as low wage individuals are forced to work long hours simply to survive^30^. In younger children and adolescents however, the relationship between SES and sleep could differ by region. Two studies found that in contrast to American and Australian children, primary schoolchildren in Hong Kong and South Korean adolescents with higher SES had shorter sleep^31,32^, possibly due to higher academic expectations by East Asian parents in the high SES group. The relatively small differences in sleep duration between the young and old in this study compared to a meta-analysis of sleep studied with polysomnography^21^ could also reflect better health and lifestyle habits in this sample. Third, only major sleep periods were analyzed, as Fitbit devices did not automatically detect sleep periods less than one hour. Shorter sleep periods could be common in older adults and East Asians likely to take naps during the day^18,33^. A fourth limitation is that certain ethnic groups reside in both regions; there could be more similarities within these groups irrespective of geographical location. In addition, there are other socio-demographic variables (e.g. urban/rural residence), lifestyle factors (e.g. diet, exercise, transportation time) and seasonal variations not accounted for here that could additionally contribute to differences in sleep schedules. Finally, feedback itself from the Fitbit app could additionally modulate behavior and findings observed particularly if expectations about ‘sleep goals’ vary by region, age group and sex.

## Conclusion

The striking regional disparity in *objectively measured* sleep duration between East Asians and Oceanians across the lifespan demonstrates the value of large scale data collection afforded by cloud connected wearable devices. While cultural factors appear to be important drivers for the shorter sleep duration in East Asians, further investigation into the reasons why this shorter sleep persists even when work obligations have receded is warranted.

## Materials and Methods

### Sleep Data

Sleep data from the period 1^st^ March 2017 to 1^st^ April 2017 contributed to this report. From an initial pool of 28,857 Fitbit users aged 15–80y, ambiguous entries from users who identified as belonging to more than one country or age group [N = 439], users who did not input sex (male/female) [N = 5] and users with less than ten weekday nights (Mon–Thu) and four weekend nights (Fri–Sat) [N = 4733] were excluded. These numbers represented the mode of the weekday and weekend frequency distributions respectively (Fig. S2) and was implemented to increase confidence in the reliability of the measures obtained while preserving a large proportion of the original dataset. The final dataset derived from 23,680 users with a total of 553,559 days of data was averaged within each user such that one user contributed two points to the dataset - one for weekday night and one for weekend night. Age was user-provided as a categorical variable in 5y bins, with the exception of the 71–80y age group. Only major sleep periods (the longest sleep episode recorded in a day) were analyzed. Bed and wake times were automatically determined based on movement data using a proprietary algorithm (https://help.fitbit.com/articles/en_US/Help_article/1314).

This study was exempt from review by the National University of Singapore Institutional Review Board, as analysis involved the use of datasets stored without identifiers. Data was provided by Fitbit Inc., and was originally collected according to Fitbit’s Terms of Service (https://www.fitbit.com/legal/terms-of-service) and Privacy Policy (https://www.fitbit.com/legal/privacy-policy).

### Statistical analysis

Analyses were conducted using SPSS 25.0 (IBM Corp., Armonk, N.Y., USA). To assess the moderating effects of age group and sex on geographical differences in sleep patterns, three-way factorial ANOVAs with between-subject factors country, age group and sex were run separately for each independent variable (sleep duration, bedtimes and wake times). Separate models were also used to inspect relationships for weekday and weekend sleep, as well as for weekday-weekend sleep extension. Where interactions were significant, pairwise comparisons among marginal means for each level of the factor of interest were contrasted. For all statistical tests, significance level was set at 0.05. However, owing to the large sample size, we focus on the magnitude of the pairwise difference, rather than on statistical significance. In addition, we also computed the proportion of users sleeping <5 h, 5–6 h, 6–7 h, 7–8 h and ≥8 h a night within each country, within each age and country subgroup, and within each sex and country subgroup, for both weekdays and weekends.

## Supplementary information

Supplementary Figures and Tables

## Data Availability

Aggregate data are available from the authors upon reasonable request.
