# Supplementary material for: Large-scale data from wearables reveal regional disparities in sleep patterns that persist across age and sex
Source: Sci Rep. 2019 Mar 4;9:3415. doi: 10.1038/s41598-019-40156-x (PMC6399225; doi:10.1038/s41598-019-40156-x)
Supplement: Supplementary file 1 — Supplementary Figures and Tables [file 41598_2019_40156_MOESM1_ESM.docx]

**­­­­Large-scale data from wearables reveal regional disparities in sleep patterns that persist across age and sex**

Ju Lynn Ong, Jesisca Tandi, Amiya Patanaik, June C. Lo and Michael W.L. Chee

Centre for Cognitive Neuroscience, Duke-NUS Medical School, Singapore 169857

**
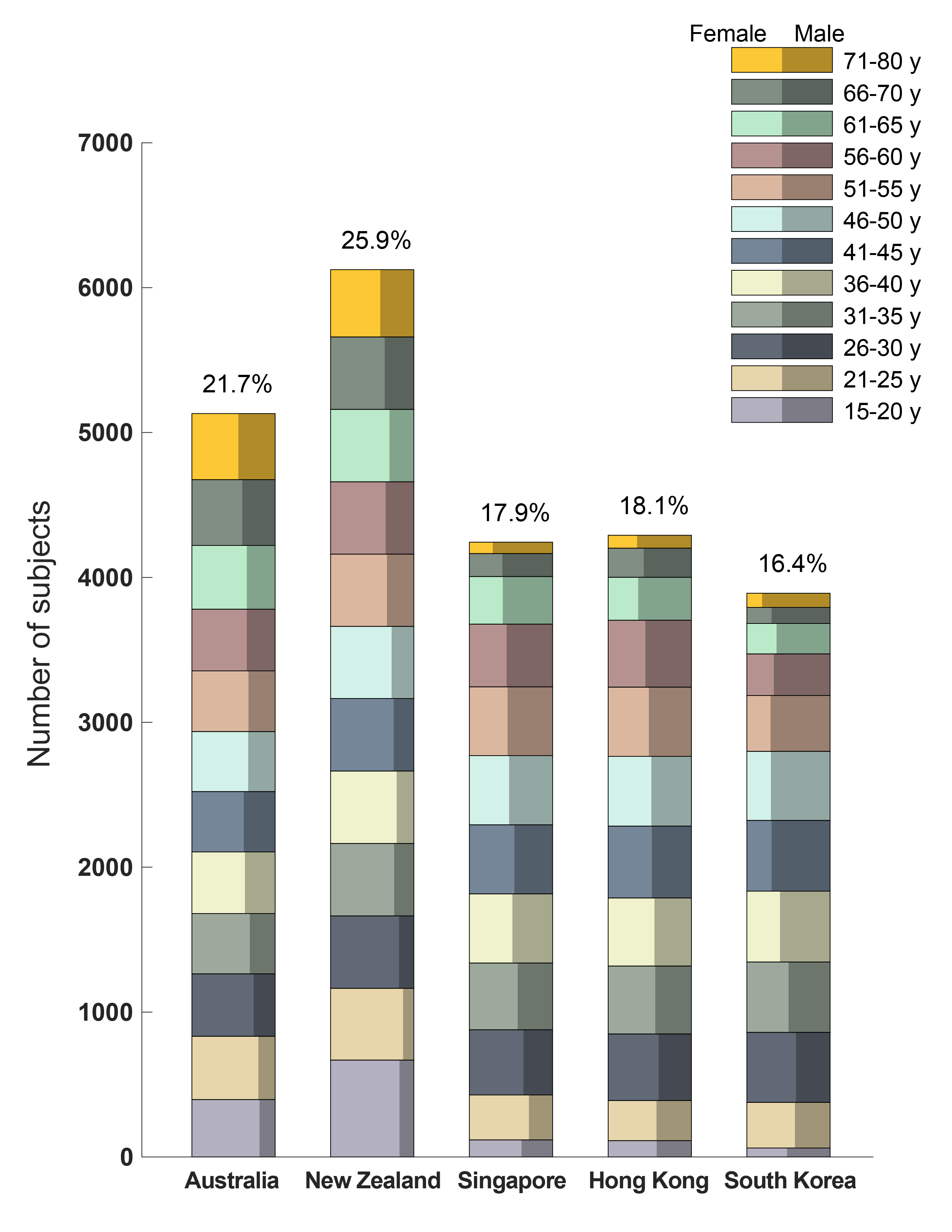
**

**Fig. S1.** **Sample characteristics by country, age group and sex.** The percentage of users by country is denoted at the top of each group of stacked bars. For each country, stacked bars represent the number of users in each age group. Shaded regions represent the proportion of males within each country and age group category.


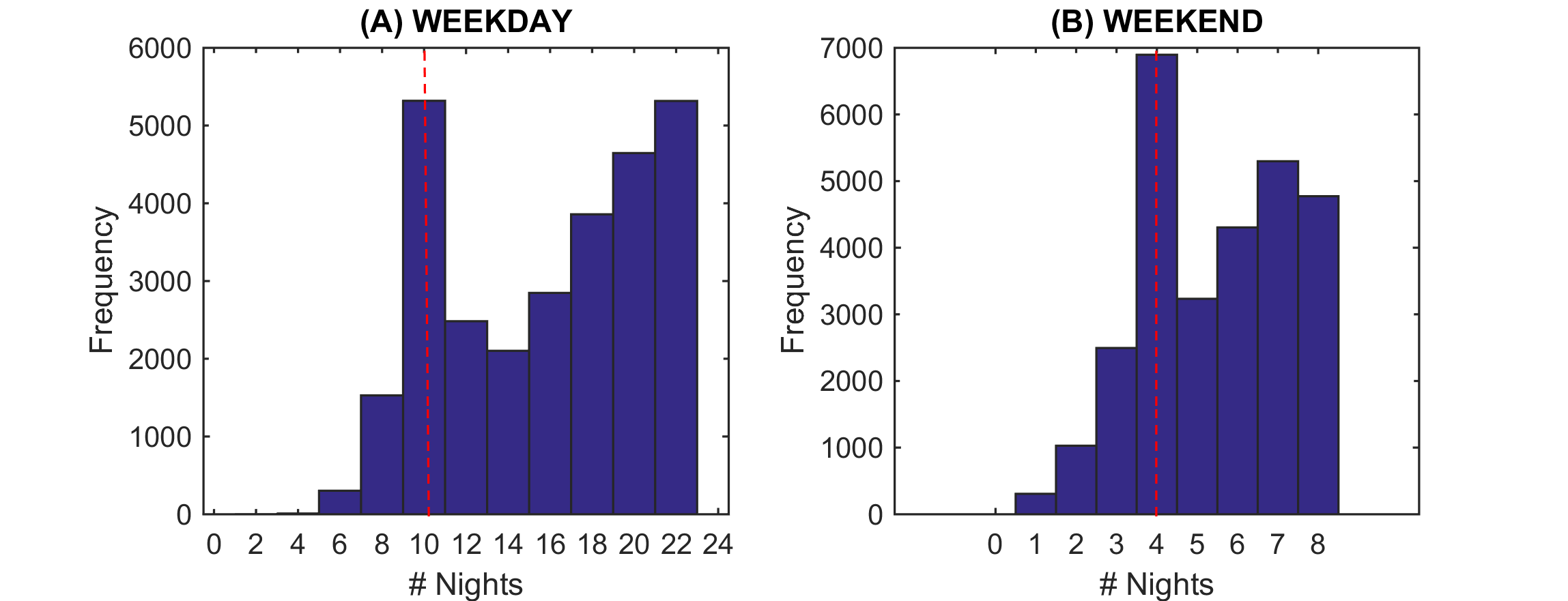


**Fig. S2. Frequency distribution of the number of nights per subject in the original dataset (N = 28857)** **for (A) weekdays and (B) weekends.** The red dotted lines represent the mode for each distribution.

**Table S1.** **Main and interaction effects of country, age group and sex on sleep patterns.** Listed values summarize results of univariate ANOVAs for main and interaction effects of country, age group and sex on weekday and weekend sleep duration, bedtimes and wake times, as well as weekday-weekend sleep extension. Bold values indicate significance at the 0.05 level.

|  | |  |  | ***F*** | ***P*** | **η_p_^2^** |
| --- | --- | --- | --- | --- | --- | --- |
| **WEEKDAY SLEEP DURATION** | |  |  |  |  |  |
| Country | |  |  | **465.19** | **<0.001** | **0.07** |
| Age Group | |  |  | **21.62** | **<0.001** | **0.01** |
| Sex | |  |  | **215.54** | **<0.001** | **0.01** |
| Country x Age Group | |  |  | **2.42** | **<0.001** | **0.00** |
| Country x Sex | |  |  | **4.10** | **0.003** | **0.00** |
| Age Group x Sex | |  |  | **2.54** | **0.003** | **0.00** |
| Country x Age Group x Sex | |  |  | 0.80 | 0.83 | 0.00 |
|  | |  |  |  |  |  |
| **WEEKDAY BEDTIME** | |  |  |  |  |  |
| Country |  |  |  | **861.49** | **<0.001** | **0.13** |
| Age Group | |  |  | **75.35** | **<0.001** | **0.03** |
| Sex |  |  |  | **82.25** | **<0.001** | **0.00** |
| Country x Age Group | |  |  | **6.00** | **<0.001** | **0.01** |
| Country x Sex | |  |  | **2.97** | **0.02** | **0.00** |
| Age Group x Sex | |  |  | **7.40** | **<0.001** | **0.00** |
| Country x Age Group x Sex | | |  | 1.13 | 0.25 | 0.00 |
|  |  |  |  |  |  |  |
| **WEEKDAY WAKE TIME** | | |  |  |  |  |
| Country |  |  |  | **244.83** | **<0.001** | **0.04** |
| Age Group | |  |  | **131.24** | **<0.001** | **0.06** |
| Sex |  |  |  | **9.48** | **0.002** | **0.00** |
| Country x Age Group | |  |  | **6.54** | **<0.001** | **0.01** |
| Country x Sex | |  |  | 0.93 | 0.45 | 0.00 |
| Age Group x Sex | |  |  | **3.27** | **<0.001** | **0.00** |
| Country x Age Group x Sex | | |  | 1.17 | 0.20 | 0.00 |
|  |  |  |  |  |  |  |
| **WEEKEND SLEEP DURATION** | | |  |  |  |  |
| Country |  |  |  | **275.90** | **<0.001** | **0.04** |
| Age Group | |  |  | **24.75** | **<0.001** | **0.01** |
| Sex |  |  |  | **172.65** | **<0.001** | **0.01** |
| Country x Age Group | |  |  | **2.69** | **<0.001** | **0.00** |
| Country x Sex | |  |  | **2.63** | **0.03** | **0.00** |
| Age Group x Sex | |  |  | **5.61** | **<0.001** | **0.00** |
| Country x Age Group x Sex | | |  | 1.23 | 0.14 | 0.00 |
|  |  |  |  |  |  |  |
| **WEEKEND BEDTIME** | |  |  |  |  |  |
| Country |  |  |  | **660.23** | **<0.001** | **0.10** |
| Age Group | |  |  | **156.20** | **<0.001** | **0.07** |
| Sex |  |  |  | **119.27** | **<0.001** | **0.01** |
| Country x Age Group | |  |  | **6.24** | **<0.001** | **0.01** |
| Country x Sex | |  |  | **4.86** | **0.001** | **0.00** |
| Age Group x Sex | |  |  | **6.81** | **<0.001** | **0.00** |
| Country x Age Group x Sex | | |  | 0.93 | 0.60 | 0.00 |
|  |  |  |  |  |  |  |
| **WEEKEND WAKE TIME** | | |  |  |  |  |
| Country |  |  |  | **252.33** | **<0.001** | **0.04** |
| Age Group | |  |  | **268.18** | **<0.001** | **0.11** |
| Sex |  |  |  | 0.67 | 0.41 | 0.00 |
| Country x Age Group | |  |  | **9.55** | **<0.001** | **0.02** |
| Country x Sex | |  |  | **2.64** | **0.03** | **0.00** |
| Age Group x Sex | |  |  | **2.05** | **0.02** | **0.00** |
| Country x Age Group x Sex | | |  | 1.00 | 0.47 | 0.00 |
|  |  |  |  |  |  |  |
| **WEEKDAY-WEEKEND SLEEP EXTENSION** | | | |  |  |  |
| Country |  |  |  | **17.76** | **<0.001** | **0.00** |
| Age Group | |  |  | **30.90** | **<0.001** | **0.01** |
| Sex |  |  |  | 0.89 | 0.35 | 0.00 |
| Country x Age Group | |  |  | **4.93** | **<0.001** | **0.01** |
| Country x Sex | |  |  | 2.33 | 0.05 | 0.00 |
| Age Group x Sex | |  |  | **2.42** | **0.005** | **0.00** |
| Country x Age Group x Sex | | |  | 1.32 | 0.08 | 0.00 |
|  | | |  |  |  |  |

**Table S2. Weekday and weekend sleep patterns by country.**

|  | **Australia** | **New Zealand** | **Singapore** | **Hong Kong** | **South Korea** |
| --- | --- | --- | --- | --- | --- |
| **Weekday Sleep** |  |  |  |  |  |
| Duration (min) * | 427.82 (0.84) | 432.77 (0.83) | 390.02 (1.06) | 395.79 (1.01) | 395.50 (1.21) |
| Bedtime (hh:mm) ^†^ | 22:59 (00:01) | 22:56 (00:01) | 23:58 (00:02) | 00:21 (00:01) | 23:58 (00:02) |
| Wake Time (hh:mm) ^‡^ | 06:43 (00:01) | 06:46 (00:01) | 07:04 (00:01) | 07:33 (00:01) | 07:07 (00:02) |
|  |  |  |  |  |  |
| **Weekend Sleep** |  |  |  |  |  |
| Duration (min) ** | 452.15 (0.99) | 460.79 (0.98) | 418.99 (1.25) | 430.45 (1.19) | 421.51 (1.44) |
| Bedtime (hh:mm) ^††^ | 23:27 (00:01) | 23:26 (00:01) | 00:28 (00:02) | 00:45 (00:02) | 00:21 (00:02) |
| Wake Time (hh:mm) ^‡‡^ | 07:37 (00:01) | 07:46 (00:01) | 08:05 (00:02) | 08:36 (00:02) | 07:59 (00:02) |
|  |  |  |  |  |  |
| **Sleep Extension (min)** ^§^ | 24.33 (0.81) | 28.02 (0.80) | 28.97 (1.02) | 34.66 (0.97) | 26.01 (1.17) |

Values are estimated means and standard errors (in parentheses) from separate univariate ANOVAs for each sleep variable taking into account the effect of age, Sex and all two- and three-way interactions.

* All pairwise comparisons were significant (P ≤ 0.001) with the exception of Hong Kong and South Korea (P = 0.85).

^†^ All pairwise comparisons were significant (P < 0.001) with the exception of Australia and New Zealand (P = 0.11), and Singapore and South Korea (P = 0.93).

^‡^ All pairwise comparisons were significant (P ≤ 0.004) with the exception of Australia and New Zealand (P = 0.70).

** All pairwise comparisons were significant (P < 0.001) with the exception of Singapore and South Korea (P = 0.19).

^††^ All pairwise comparisons were significant (P < 0.001) with the exception of Australia and New Zealand (P = 0.10), and Singapore and South Korea (P = 0.12).

^‡‡^ All pairwise comparisons were significant (P ≤ 0.007).

^§^ Sleep extension was computed using weekend-weekday sleep duration. All pairwise comparisons were significant (P ≤ 0.001) with the exception of Australia and South Korea (P = 0.24), New Zealand and Singapore (P = 0.47), New Zealand and South Korea (P = 0.16) and Singapore and South Korea (P = 0.06).

**Table S3. Country differences in weekday sleep duration (separated by age group).**

|  | **Australia** | **New Zealand** | **Singapore** | **Hong Kong** | **South Korea** |
| --- | --- | --- | --- | --- | --- |
| **15-20y** |  |  |  |  |  |
| Australia | - | -0.59 | 52.27*** | 54.36*** | 63.50*** |
| New Zealand | 0.59 | - | 52.87*** | 54.95*** | 64.09*** |
| Singapore | -52.27*** | -52.87*** | - | 2.09 | 11.22 |
| Hong Kong | -54.36*** | -54.95*** | -2.09 | - | 9.13 |
| South Korea | -63.50*** | -64.09*** | -11.22 | -9.13 | - |
|  |  |  |  |  |  |
| **21-25y** |  |  |  |  |  |
| Australia | - | -2.60 | 45.27*** | 40.88*** | 35.61*** |
| New Zealand | 2.60 | - | 47.87*** | 43.48*** | 38.21*** |
| Singapore | -45.27*** | -47.87*** | - | -4.39 | -9.65* |
| Hong Kong | -40.88*** | -43.48*** | 4.39 | - | -5.26 |
| South Korea | -35.61*** | -38.21*** | 9.65* | 5.26 | - |
|  |  |  |  |  |  |
| **26-30y** |  |  |  |  |  |
| Australia | - | -11.75** | 31.89*** | 32.74*** | 20.31*** |
| New Zealand | 11.75** | - | 43.65*** | 44.49*** | 32.06*** |
| Singapore | -31.89*** | -43.65*** | - | 0.84 | -11.58** |
| Hong Kong | -32.74*** | -44.49*** | -0.84 | - | -12.42** |
| South Korea | -20.31*** | -32.06*** | 11.58** | 12.42** | - |
|  |  |  |  |  |  |
| **31-35y** |  |  |  |  |  |
| Australia | - | 0.38 | 30.57*** | 33.51*** | 30.26*** |
| New Zealand | -0.38 | - | 30.19*** | 33.13*** | 29.88*** |
| Singapore | -30.57*** | -30.19*** | - | 2.94 | -0.31 |
| Hong Kong | -33.51*** | -33.13*** | -2.94 | - | -3.25 |
| South Korea | -30.26*** | -29.88*** | 0.31 | 3.25 | - |
|  |  |  |  |  |  |
| **36-40y** |  |  |  |  |  |
| Australia | - | -2.97 | 29.92*** | 32.69*** | 28.80*** |
| New Zealand | 2.97 | - | 32.89*** | 35.67*** | 31.77*** |
| Singapore | -29.92*** | -32.89*** | - | 2.77 | -1.12 |
| Hong Kong | -32.69*** | -35.67*** | -2.77 | - | -3.9 |
| South Korea | -28.80*** | -31.77*** | 1.12 | 3.9 | - |
|  |  |  |  |  |  |
| **41-45y** |  |  |  |  |  |
| Australia | - | -6.59 | 32.74*** | 27.04*** | 31.95*** |
| New Zealand | 6.59 | - | 39.33*** | 33.63*** | 38.55*** |
| Singapore | -32.74*** | -39.33*** | - | -5.70 | -0.78 |
| Hong Kong | -27.04*** | -33.63*** | 5.70 | - | 4.92 |
| South Korea | -31.95*** | -38.55*** | 0.78 | -4.92 | - |
|  |  |  |  |  |  |
| **46-50y** |  |  |  |  |  |
| Australia | - | -13.49** | 29.65*** | 25.85*** | 25.88*** |
| New Zealand | 13.49** | - | 43.13*** | 39.33*** | 39.36*** |
| Singapore | -29.65*** | -43.13*** | - | -3.80 | -3.77 |
| Hong Kong | -25.85*** | -39.33*** | 3.80 | - | 0.03 |
| South Korea | -25.88*** | -39.36*** | 3.77 | -0.03 | - |
|  |  |  |  |  |  |
| **51-55y** |  |  |  |  |  |
| Australia | - | -2.31 | 39.12*** | 28.69*** | 35.64*** |
| New Zealand | 2.31 | - | 41.43*** | 31.00*** | 37.95*** |
| Singapore | -39.12*** | -41.43*** | - | -10.43** | -3.48 |
| Hong Kong | -28.69*** | -31.00*** | 10.43** | - | 6.95 |
| South Korea | -35.64*** | -37.95*** | 3.48 | -6.95 | - |
|  |  |  |  |  |  |
| **56-60y** |  |  |  |  |  |
| Australia | - | -4.75 | 35.94*** | 25.93*** | 22.87*** |
| New Zealand | 4.75 | - | 40.69*** | 30.68*** | 27.63*** |
| Singapore | -35.94*** | -40.69*** | - | -10.01** | -13.07** |
| Hong Kong | -25.93*** | -30.68*** | 10.01** | - | -3.06 |
| South Korea | -22.87*** | -27.63*** | 13.07** | 3.06 | - |
|  |  |  |  |  |  |
| **61-65y** |  |  |  |  |  |
| Australia | - | -0.60 | 43.19*** | 22.23*** | 28.92*** |
| New Zealand | 0.60 | - | 43.80*** | 22.84*** | 29.52*** |
| Singapore | -43.19*** | -43.80*** | - | -20.96*** | -14.27** |
| Hong Kong | -22.23*** | -22.84*** | 20.96*** | - | 6.69 |
| South Korea | -28.92*** | -29.52*** | 14.27** | -6.69 | - |
|  |  |  |  |  |  |
| **66-70y** |  |  |  |  |  |
| Australia | - | -6.23 | 46.70*** | 29.22*** | 29.89*** |
| New Zealand | 6.23 | - | 52.93*** | 35.45*** | 36.12*** |
| Singapore | -46.70*** | -52.93*** | - | -17.48** | -16.81* |
| Hong Kong | -29.22*** | -35.45*** | 17.48** | - | 0.67 |
| South Korea | -29.89*** | -36.12*** | 16.81* | -0.67 | - |
|  |  |  |  |  |  |
| **71-80y** |  |  |  |  |  |
| Australia | - | -7.83* | 36.37*** | 31.19*** | 34.24*** |
| New Zealand | 7.84* | - | 44.20*** | 39.03*** | 42.08*** |
| Singapore | -36.37*** | -44.20*** | - | -5.18 | -2.12 |
| Hong Kong | -31.19*** | -39.03*** | 5.18 | - | 3.06 |
| South Korea | -34.24*** | -42.08*** | 2.12 | -3.055 | - |

Comparisons are statistically significant at *P < 0.05, **P < 0.01, ***P < 0.001. Values represent differences of marginal means between countries listed in each row and column.

**Table S4.** **Country differences in weekend sleep duration (separated by age group).**

|  | **Australia** | **New Zealand** | **Singapore** | **Hong Kong** | **South Korea** |
| --- | --- | --- | --- | --- | --- |
| **15-20y** |  |  |  |  |  |
| Australia | - | 0.50 | 17.99* | 20.21** | 35.18*** |
| New Zealand | -0.50 | - | 17.49* | 19.71** | 34.68*** |
| Singapore | -17.99* | -17.49* | - | 2.22 | 17.19* |
| Hong Kong | -20.21*** | -19.71*** | -2.22 | - | 14.97 |
| South Korea | -35.18*** | -34.68*** | -17.19* | -14.97 | - |
|  |  |  |  |  |  |
| **21-25y** |  |  |  |  |  |
| Australia | - | -10.57 | 30.75*** | 21.89*** | 20.50*** |
| New Zealand | 10.57 | - | 41.32*** | 32.46*** | 31.07*** |
| Singapore | -30.75*** | -41.32*** | - | -8.86 | -10.25 |
| Hong Kong | -21.89*** | -32.46*** | 8.86 | - | -1.39 |
| South Korea | -20.50*** | -31.07*** | 10.25 | 1.39 | - |
|  |  |  |  |  |  |
| **26-30y** |  |  |  |  |  |
| Australia | - | -7.44 | 22.15*** | 13.36** | 16.60*** |
| New Zealand | 7.44 | - | 29.59*** | 20.80*** | 24.04*** |
| Singapore | -22.15*** | -29.59*** | - | -8.79* | -5.55 |
| Hong Kong | -13.36** | -20.80*** | 8.79* | - | 3.24 |
| South Korea | -16.60*** | -24.04*** | 5.55 | -3.24 | - |
|  |  |  |  |  |  |
| **31-35y** |  |  |  |  |  |
| Australia | - | -5.87 | 20.33*** | 11.20* | 21.73*** |
| New Zealand | 5.87 | - | 26.20*** | 17.07*** | 27.59*** |
| Singapore | -20.33*** | -26.20*** | - | -9.14* | 1.39 |
| Hong Kong | -11.20* | -17.07*** | 9.14* | - | 10.53* |
| South Korea | -21.73*** | -27.59*** | -1.39 | -10.53* | - |
|  |  |  |  |  |  |
| **36-40y** |  |  |  |  |  |
| Australia | - | -1.66 | 27.35*** | 23.93*** | 25.79*** |
| New Zealand | 1.66 | - | 29.01*** | 25.59*** | 27.45*** |
| Singapore | -27.35*** | -29.01*** | - | -3.41 | -1.55 |
| Hong Kong | -23.93*** | -25.59*** | 3.41 | - | 1.86 |
| South Korea | -25.79*** | -27.45*** | 1.55 | -1.86 | - |
|  |  |  |  |  |  |
| **41-45y** |  |  |  |  |  |
| Australia | - | -5.90 | 35.16*** | 15.96*** | 32.48*** |
| New Zealand | 5.90 | - | 41.05*** | 21.86*** | 38.38*** |
| Singapore | -35.16*** | -41.05*** | - | -19.19*** | -2.68*** |
| Hong Kong | -15.96*** | -21.86*** | 19.19*** | - | 16.52*** |
| South Korea | -32.48*** | -38.38*** | 2.68 | -16.52*** | - |
|  |  |  |  |  |  |
| **46-50y** |  |  |  |  |  |
| Australia |  | -16.91*** | 26.84*** | 19.02*** | 29.06*** |
| New Zealand | 16.91*** |  | 43.74*** | 35.93*** | 45.97*** |
| Singapore | -26.84*** | -43.74*** |  | -7.81 | 2.22 |
| Hong Kong | -19.02*** | -35.93*** | 7.81 |  | 10.03* |
| South Korea | -29.06*** | -45.97*** | -2.22 | -10.03* |  |
|  |  |  |  |  |  |
| **51-55y** |  |  |  |  |  |
| Australia | - | -5.66 | 40.98*** | 25.74*** | 38.58*** |
| New Zealand | 5.66 | - | 46.64*** | 31.40*** | 44.24*** |
| Singapore | -40.98*** | -46.64*** | - | -15.24*** | -2.40 |
| Hong Kong | -25.74*** | -31.40*** | 15.24*** | - | 12.84** |
| South Korea | -38.58*** | -44.24*** | 2.40 | -12.84** | - |
|  |  |  |  |  |  |
| **56-60y** |  |  |  |  |  |
| Australia | - | -10.33* | 42.50*** | 28.24*** | 35.00*** |
| New Zealand | 10.33* | - | 52.83*** | 38.57*** | 45.33*** |
| Singapore | -42.50*** | -52.83*** | - | -14.26** | -7.50 |
| Hong Kong | -28.24*** | -38.57*** | 14.26** | - | 6.76 |
| South Korea | -35.00*** | -45.33*** | 7.50 | -6.76 | - |
|  |  |  |  |  |  |
| **61-65y** |  |  |  |  |  |
| Australia | - | -14.23** | 48.58*** | 28.30*** | 35.80*** |
| New Zealand | 14.23** | - | 62.81*** | 42.53*** | 50.03*** |
| Singapore | -48.58*** | -62.81*** | - | -20.29*** | -12.78* |
| Hong Kong | -28.30*** | -42.53*** | 20.29*** | - | 7.50 |
| South Korea | -35.80*** | -50.03*** | 12.78* | -7.50 | - |
|  |  |  |  |  |  |
| **66-70y** |  |  |  |  |  |
| Australia | - | -12.79** | 47.60*** | 25.64*** | 36.75*** |
| New Zealand | 12.79** | - | 60.38*** | 38.43*** | 49.54*** |
| Singapore | -47.60*** | -60.38*** | - | -21.95** | -10.84 |
| Hong Kong | -25.64*** | -38.43*** | 21.95** | - | 11.11 |
| South Korea | -36.75*** | -49.54*** | 10.84 | -11.11 | - |
|  |  |  |  |  |  |
| **71-80y** |  |  |  |  |  |
| Australia | - | -12.86** | 37.72*** | 26.81*** | 40.21*** |
| New Zealand | 12.86** | - | 50.57*** | 39.67*** | 53.07*** |
| Singapore | -37.72*** | -50.57*** | - | -10.90 | 2.49 |
| Hong Kong | -26.81** | -39.67*** | 10.90 | - | 13.39 |
| South Korea | -40.21*** | -53.07*** | -2.49 | -13.39 | - |
|  |  |  |  |  |  |

Comparisons are statistically significant at *P < 0.05, **P < 0.01, ***P < 0.001. Values represent differences of marginal means between countries listed in each row and column.

**Table S5. Country differences in weekday sleep duration (separated by sex).**

|  | **Australia** | **New Zealand** | **Singapore** | **Hong Kong** | **South Korea** |
| --- | --- | --- | --- | --- | --- |
| **Females** |  |  |  |  |  |
| Australia | - | -2.93* | 43.07*** | 34.83*** | 33.48*** |
| New Zealand | 2.93* | - | 46.00*** | 37.76*** | 36.40*** |
| Singapore | -43.07*** | -46.00*** | - | -8.24*** | -9.60*** |
| Hong Kong | -34.83*** | -37.76*** | 8.24*** | - | -1.35 |
| South Korea | -33.48*** | -36.40*** | 9.60*** | 1.35 | - |
|  |  |  |  |  |  |
| **Males** |  |  |  |  |  |
| Australia | - | -6.97*** | 32.53*** | 29.22*** | 31.17*** |
| New Zealand | 6.97*** | - | 39.50*** | 36.19*** | 38.13*** |
| Singapore | -32.53*** | -39.50*** | - | -3.31 | -1.36 |
| Hong Kong | -29.22*** | -36.19*** | 3.31 | - | 1.95 |
| South Korea | -31.17*** | -38.13*** | 1.36 | -1.95 | - |

Comparisons are statistically significant at *P < 0.05, **P < 0.01, ***P < 0.001. Values represent differences of marginal means between countries listed in each row and column.

**Table S6.** **Country differences in weekend sleep duration (separated by sex).**

|  | **Australia** | **New Zealand** | **Singapore** | **Hong Kong** | **South Korea** |
| --- | --- | --- | --- | --- | --- |
| **Females** |  |  |  |  |  |
| Australia | - | -7.99*** | 37.63*** | 21.94*** | 33.49*** |
| New Zealand | 7.99*** | - | 45.63*** | 29.93*** | 41.49*** |
| Singapore | -37.63*** | -45.63*** | - | -15.70*** | -4.14 |
| Hong Kong | -21.94*** | -29.93*** | 15.70*** | - | 11.56*** |
| South Korea | -33.49*** | -41.49*** | 4.14 | -11.56*** | - |
|  |  |  |  |  |  |
| **Males** |  |  |  |  |  |
| Australia | - | -9.29*** | 28.69*** | 21.45*** | 27.79*** |
| New Zealand | 9.29*** | - | 37.98*** | 30.74*** | 37.08*** |
| Singapore | -28.69*** | -37.98*** | - | -7.24** | -0.90 |
| Hong Kong | -21.45*** | -30.74*** | 7.24** | - | 6.34** |
| South Korea | -27.79*** | -37.08*** | 0.90 | -6.34** | - |

Comparisons are statistically significant at *P < 0.05, **P < 0.01, ***P < 0.001. Values represent differences of marginal means between countries listed in each row and column.
